# Vitamin Deficiency or Insufficiency Hypothesis: A Global Health Crisis of Biological Influence Towards Development of Antimicrobial Resistant Strains. A Perspective View

**DOI:** 10.1002/hsr2.72121

**Published:** 2026-03-16

**Authors:** Harold L. Mashauri

**Affiliations:** ^1^ Department of Clinical Physiology, School of Medicine KCMC University Moshi Tanzania; ^2^ Department of Epidemiology and Biostatistics, School of Public Health KCMC University Moshi Tanzania; ^3^ Department of Internal Medicine, School of Medicine KCMC University Moshi Tanzania

**Keywords:** AMR evolution, AMR genesis, antimicrobial resistance, climate change, global health, infectious diseases, nutrition, vitamins

## Abstract

**Background and Aim:**

Antimicrobial resistance (AMR) has been a growing crisis of economic, clinical, and public health concern for years. Different mechanisms of why and how AMR develops have been suggested and studied. Vitamins' immunological and genomic influence towards the evolution of resistant microbial agents makes them among the potential candidates to be examined when questioning the genesis and evolution of the AMR crisis. Unfortunately, this area is less explored. This perspective article aims at highlighting the potential role of vitamins towards the global AMR crisis development and suggesting potential hypothetical biological link and areas of further studies.

**Findings:**

Antimicrobial effect and reversal genomic influence of different vitamins among resistant microbial strains suggest a potential biological link in terms of resistant genes genesis and suppression when subjected to environments with some vitamins' concentrations below optimal levels and vice versa. Inadequate intake of fruits and vegetables along the effects of climate changes on nutritional quality and quantity of crop products are among the key potential driving factors towards AMR crisis globally. Since different vitamins like vitamin C play a key role towards DNA stability by preventing and controlling DNA mutations, the potential biological influence of different vitamins towards AMR development is worth consideration for further exploration and should not be underestimated.

**Conclusion:**

Screening and supplementing strategies of specific vitamins of interest among resistant microbial‐ infected patients should be studied further to understand the biological link between specific vitamin deficiency or insufficiency levels and the development of specific antimicrobial‐resistant strains. The role and influence of climate change on this biological link should also be explored in detail from a health perspective. Furthermore, different global health interventions should be implemented to increase the global intake of fruits and vegetables as one of the potential sustainable approaches towards prevention and control of AMR crisis, especially in developing countries.

## 1 Introduction

Antimicrobial Resistance (AMR) has been a growing crisis of economic, clinical, and public health concern for years now [1, 2, 3]. The rational use of different antimicrobial agents alongside infection prevention and control programs is among the strategies which have been advocated to address the issue in terms of practice and awareness among both healthcare workers and the general population. Different mechanisms of why and how AMR develops have been suggested, including the development of resistant genes secondary to improper use of antimicrobial agents, especially antibiotics [2, 4]. The expression of these genes enables microorganisms to modify the drug targets, reduce the drug uptake, inactivate antimicrobial agents, and increase their efflux [5]. These mechanisms can be natural or acquired from other resistant microorganisms [5]. Moreover, these mechanisms have caught the attention of both clinical and public health scientists in searching for effective interventions to combat the crisis.

The immunological and genomic influence of vitamins on the evolution of different resistant microbial agents alongside their antimicrobial effect make them among the most potential areas to be examined when questioning the genesis and evolution of the AMR crisis [6]. Unfortunately, the role of different vitamin deficiencies or insufficiencies towards the development of different resistant microbial strains has not been much expounded.

Although there is limited recent data on deficiency and insufficiency of different vitamins globally among the general population, several studies have shown significant deficiencies and insufficiencies of different vitamins like vitamin A, B, C, and D among hospitalized patients, whereby some showed a significant association between vitamin deficiencies or insufficiencies with increased mortality from infectious diseases which was reduced upon vitamin supplementation [7, 8, 9, 10, 11, 12, 13, 14, 15, 16]. While it remains unclear whether such vitamin deficiencies or insufficiencies were due to the illness severity or vice versa, the immune‐protective and antimicrobial effect of different vitamins suggests the former association. Although the prevalence of hypovitaminosis in the general population is approximated to be 7%, the prevalence of vitamin C deficiency among hospitalized patients is 47.3% [17].

Globally, it has been recently shown that the intake of both vegetables and fruits, which are among the richest sources of different vitamins, was found to be below the recommendations in 88% of countries, particularly in developing countries [18, 19, 20]. Vitamin C hypovitaminosis and deficiency have been found to be more common and prevalent in Low and Middle‐Income Countries (LMICs) [21]. Moreover, the consumption of fruits and vegetables was found to be low in over 80% of individuals aged greater than 15 years in LMICs, with the simultaneous burden of AMR crisis being more pronounced in these countries compared to developed countries [22, 23, 24]. This AMR burden in LMICs has been associated with an increase in bloodstream infection mortality rates by 58%, a doubling of the odds of ICU admissions, a prolongation of hospital stays by a week, and an increase in direct medical costs of about $12,000 per case [25].

This article suggests a literature‐supported hypothesized biological link between vitamin deficiencies or insufficiencies and the genesis and evolution of the AMR crisis globally. It aims to highlight to both clinical and public health scientists the potential biological areas which might need further studies as the world is searching for sustainable and effective prevention and control interventions towards the fight against AMR crisis.

## 2 Climate Change and Vitamin Deficiency or Insufficiency

Climate change, especially in terms of increased atmospheric CO_2_ levels and global warming, significantly affects food security and safety [26, 27, 28, 29]. An increase in global surface temperature leads to extreme weather, which causes drought, increased biotic stressors, heat waves, and flooding [26]. This reduces food production and distribution, hence impacting food availability, especially in areas like developing countries, which depend highly on agricultural products as the source of food [27, 28]. Moreover, this affects the nutritional quality and quantity of harvested food products [27, 30].

While the quality and levels of different nutrients like proteins and minerals in various crops have been found to decline in response to climatic changes, the effect on both quality and quantity of different vitamins is less explored [27, 28, 30]. Nevertheless, since most vitamins especially water‐soluble vitamins like vitamin C and B are more heat sensitive compared to most minerals and macronutrients like proteins, their nutritional quality and quantity might be more affected [31, 32]. Furthermore, several studies have demonstrated the decline in both vegetable and fruit yield as a result of climate change [28, 29]. These findings also revealed a significant decrease in the level of their micronutrient content. This necessitates the need to implement agricultural practices that preserve and enhance the nutritional quality and quantity of crops amidst the effects of climate change [33].

## 3 Vitamins and AMR

### 3.1 Antimicrobial and Genomic Influence of Vitamins on Resistant Microbial Strains

Different vitamins like vitamin A, B, C, D and E have been shown to possess antimicrobial effects against viral, parasitic, bacterial and fungal strains independently or in combination with various classes of antimicrobial drugs [6]. This antimicrobial effect has proven to be effective even against multi‐drug resistance strains [6].

Surprisingly, vitamins have also demonstrated a significant genomic influence towards different antimicrobial‐resistant strains by terminating and/or reversing the expression of resistant genes [6]. Studies have documented such effects for vitamins B and C [6]. Additionally, vitamin C has been shown to possess a protective effect against DNA instability and in the prevention and control of DNA mutations [34, 35, 36].

### 3.2 Host–Pathogen Interaction and the Role of Vitamins

The host–pathogen interaction profile determines the likelihood of infection establishment and its severity [37]. The host immune system is activated whenever there is pathogenic invasion through inflammatory cytokines and chemokine cascades. In return, pathogens deploy several microbiological responses to escape, modulate, or bypass the host's immunity [37].

Different vitamins have been found to modulate and enhance the effectiveness of the host's immune system responses towards different infections [6]. Even though different pathogens utilize several vitamins for various essential cellular activities, including protein synthesis, optimization of these vitamins in the host's environment might improve the host's resistance to infection [38]. While optimal serum levels of different vitamins enhance immune clearance of different infections, some infections might lead to low levels of such vitamins, given the role of some of the vitamins as counteracting antioxidants during infectious inflammatory processes alongside pathogens' and host's cellular and immune system vitamins requirements, respectively [6, 38].

### 3.3 Vitamin Deficiency or Insufficiency‐Induced AMR Development Hypothesis

Given the genomic influence of several vitamins, especially water‐soluble vitamins, on the genetic stability of different microbial agents and the fact that suppression and reversal of resistant gene expression effect upon exposure to some vitamins has been documented in some studies [6], the following can be hypothesized; there is a required optimal serum concentration level of each vitamin that must be maintained throughout every physiological system, including both the human body and within microorganisms. This hypothesis assumes the established serum concentration levels or values for different vitamins to be the optimal concentration level of the respective vitamin.

During infection, if the serum vitamin levels in either the microorganism, the infected biological system, like the human body or both are within optimal ranges, microbial agents remain genetically stable and sensitive to their designated antimicrobial drugs. Vitamins' optimal levels prevent or inhibit the expression of resistant genes among the infecting microorganisms. This effect is more pronounced with some specific types of vitamins.

In case of infection but with some vitamins' serum concentration above the optimal levels in the human body, such levels tend to act as endogenous antimicrobial agents whereby in combination with body's immunity, it can lead to clearance of infection even before the use of exogenous antimicrobial drugs. The higher the concentration, the greater the endogenous antimicrobial effect. This might explain why some studies have demonstrated the independent antimicrobial effect of some vitamins in vitro [6]. On the other hand, if the serum concentrations of some vitamins are below the optimal levels during infections, such levels tend to make microbial agents unstable genetically, and different mutations are likely to occur, which lead to the expression of resistant genes. These levels turn on the expression of such genes. The lower the concentration, the higher the extent of mutations and the more drugs of different classes the microorganism becomes resistant to.

Microbial agents acquire resistance secondary to various mutations in response to exposure to an environment with vitamin concentrations below their optimal requirement for a long time. Such resistant strains can spread from one host to another and can cross‐transmit such genes among themselves. When a drug‐sensitive microbial agent is subjected to an environment with vitamins' concentration below optimal levels, it will remain sensitive (to exogenous antimicrobial agents) as long as its cellular stores are still sufficient enough to ensure its genetic stability. However, with time, these vitamin stores become depleted, and since both the internal and external environments are depleted with such vitamins, the microbial agent will become unstable genetically and can develop different mutations in response to different triggers like the immune system, drug attacks, and other noxious triggers in the environment. These mutations can be of protective or suicidal effect on the microbial agents. With a protective effect, it ensures the survival of such microbial agents even in harsh environments.

There are different ways in which resistant microbial strains (with resistant gene expression) can develop based on this hypothesis. When a non‐resistant microbial strain is exposed to a host with optimal or above optimal concentration levels of some vitamins, it will remain sensitive and susceptible to its designated antimicrobial agents. However, when it is subjected to the host with suboptimal vitamin concentration levels for some time, it will develop resistance through the expression of various resistant genes and become resistant to its designated antimicrobial agents in the long run. When such a host is supplemented with specific deficient or insufficient vitamins to the extent of raising their concentrations to the optimal or above optimal levels for some time, the expression of resistance genes will likely be reversed, and subside, and with time, the strain will regain its susceptibility to its designated antimicrobial agents. This implies that hosts infected with a resistant strain should be tested for specific vitamin deficiency or insufficiency, and antimicrobial agents of choice should be co‐administered with a specific deficient or insufficient vitamin. Another viable option is that such hosts can be subjected to the known deficient or insufficient vitamin initially for some time before initiating the antimicrobial agents.

While antimicrobial resistance can be facilitated by the irrational use of antimicrobial agents, it is primarily secondary to exposure of infectious microorganisms to an environment with suboptimal vitamin concentrations, regardless of how frequently the host uses antimicrobial agents irrationally. This might be why some studies have shown a synergetic antimicrobial effect of some antimicrobial agents in combination with some specific vitamins, even against microbes that were initially resistant to their respective drugs [6].

Water soluble vitamin deficiency especially vitamin C is likely to play a great role in this biological link since such vitamins never get stored in most hosts including human beings. If these vitamins are not consumed routinely, mild to moderate deficiency or insufficiency might occur within a few days. Several studies reported the reversal of genotypic resistant gene expression among resistant bacterial clinical isolates post‐vitamin C exposure [6].

## 4 Discussion

Declining in food production, distribution, and nutritional quality, including the expected levels of different micronutrients, endangers global health by increasing the risk and prevalence of malnutrition‐related conditions like vitamin deficiencies or insufficiencies [26, 27, 28, 30]. This might have confounded the observation that vitamin insufficiencies or deficiencies might be prevalent secondary to the intake of fruits and vegetables globally being below the recommended intake globally, especially in developing countries [18, 19, 20]. Considering the proposed hypothesis above, reduced fruits and vegetables production along with poor nutritional quality and quantity in response to climate changes might be the predisposing and facilitating factors towards AMR evolution. Furthermore, the impact on the nutritional quality and quantity of essential micronutrients like vitamins might also be affecting the rest of global vegetation, hence, predisposing both animals and microorganisms to a food chain or environment with vitamins below the optimal levels. This can further precipitate the AMR crisis.

While the possible mechanisms involved in the development of antimicrobial resistance including the role of frequent misuse of antimicrobial agents have been well studied and documented, it is still not clearly understood what triggers such mechanisms apart from just being considered as biological survival mechanisms [5]. Moreover, given the complexity in terms of inter‐ and intra‐dependency of these mechanisms, the AMR crisis is still prevalent despite the development of therapeutics that target these mechanisms [5].

The antimicrobial effect and genomic influence of different vitamins towards various microbial strains suggest a biological link in terms of resistant gene evolution among microbial strains when subjected to an environment with suboptimal concentration levels of some vitamins [6]. This effect can be reversed when such vitamins' concentration levels are adjusted to or above optimal levels. Moreover, the hypothesized biological link above suggests that the increase in AMR crisis magnitude globally reflects the increasing prevalence of different vitamin deficiencies or insufficiencies among the general population, particularly the infected ones [18, 19, 20, 21, 22, 23, 24, 25]. This link might be due to the potential role of different vitamins in the prevention and control of cellular mutations at different cellular proliferation levels, specifically among microorganisms [34, 35, 36].

The misuse of different antimicrobial agents might be among the key drivers towards AMR development based on the established understanding that multiple or repeated exposure to antimicrobial agents, especially below lethal doses, enables pathogens to adapt and develop defensive mechanisms [2, 4]. Still, the theory linking vitamin deficiencies or insufficiencies and AMR development, as explained above, is interwoven within this concept since among the defensive mechanisms pathogens develop in response or as part of adaptation upon multiple exposures to antimicrobial agents is increased expression of resistant genes which code for proteins which increase the number and efficiency of microbial defensive pathways like efflux pumps [2, 4]. The proposed hypothesis explains the potential trigger for AMR development mechanisms instead of simply considering it to be a natural survival mechanism among microorganisms [5]. This might explain why different studies have demonstrated the protective role of vitamins to such adaptation through prevention and control of resistant gene expression with the enhancement of antibiotics' effectiveness when co‐administered simultaneously, even to resistant strains that such antibiotics were not sensitive to at first [6].

The effectiveness of the newly developed antimicrobial agents might still be challenged by the vitamin deficiencies or insufficiencies in the host with time. While vitamin deficiencies or insufficiencies status may be necessary and sufficient for AMR development, multiple antimicrobial exposures, even below lethal doses may not be sufficient or necessary for AMR development in the presence of optimal levels of different vitamins, especially during host–pathogen interaction.

The fact that some studies have reported the suppression and reversal of resistant genes when resistant microorganisms were subjected to optimal amount or levels of vitamins like vitamin C, also suggests that resistance of different microbial strains in different hosts can potentially be reversed by supplementing such hosts with the specific deficient vitamin [6]. This can also lead to the so‐called “Return of Drug Sensitivity” (RoDS) among such resistant microorganisms. This is whereby a certain microbial strain which was initially resistant to the respective antimicrobial drug, becomes sensitive to the same drug after some period of time due to different reasons including the reversal of resistant gene expression following the biological intervention of interest like post vitamin exposure in a given time.

While host–pathogen interactions can deplete or reduce the host's intracellular vitamins' stores or serum levels, respectively, due to their increased consumption by both the host's immune system and the pathogen's cellular processes, infection with resistant strains can worsen the demands of such vitamins [6, 7, 8, 9, 10, 11, 12, 13, 14, 15, 16, 17, 38]. This can be reflected in developing countries where the intake of fruits and vegetables is low, with high prevalence of hypovitaminosis and high burden of AMR concurrently [18, 19, 20, 21, 22, 23, 24].

## 5 Conclusion and Recommendation

Evidence on the antimicrobial and genomic influence and effect of different vitamins, even on multi‐drug resistant strains, has been rapidly accumulating recently. Given the critical role of different vitamins, especially water‐soluble vitamins like vitamin C is of paramount importance in DNA stability in terms of prevention and control of DNA mutations, the potential biological influence of various vitamins towards AMR development is worth consideration for further exploration and should not be underestimated.

It should also be studied whether patients diagnosed with resistant infectious strains should or should not be screened and supplemented with specific vitamins of interest in case of deficiency or insufficiency, so as to improve drug sensitivity in terms of return of drug sensitivity (RoDS). Water‐soluble vitamins like vitamin C should be given priority in screening and supplementation, given their potential role in the prevention and control of different mutations at the cellular level.

The role of climate change effects towards AMR development is vividly appreciated, given their consequences on food security, nutritional quality and quantity, and potential theoretical role in AMR evolution. Collaborative efforts among relevant global key stakeholders should be deployed against climate change driving factors using one health approach. Moreover, countries should invest in studying, designing, adapting, and deploying sustainable and innovative approaches towards the preservation of nutritional quality and quantity in agricultural food products amidst the ongoing climate change crisis.

Furthermore, studies should be done to determine the magnitude of deficiency or insufficiency of different vitamins among patients diagnosed with different infectious diseases and explore in detail the biological link between specific deficiency or insufficiency levels and the development of antimicrobial‐resistant strains. Different global health interventions should be implemented to increase the global intake of fruits and vegetables as one of the potential sustainable approaches towards prevention and control of AMR crisis especially, in developing countries.

## Author Contributions

H.L.M. conceptualized the manuscript, administered, and supervised the project. H.L.M. did data curation, wrote and edited the first draft of the manuscript, and reviewed and approved the final draft for publication.

## Funding

The author received no specific funding for this work.

## Ethics Statement

The author has nothing to report.

## Consent

The author has nothing to report.

## Conflicts of Interest

The author declares no conflicts of interest.

## Transparency Statement

The lead author, Harold L. Mashauri, affirms that this manuscript is an honest, accurate, and transparent account of the study being reported; that no important aspects of the study have been omitted; and that any discrepancies from the study as planned (and, if relevant, registered) have been explained.

## Data Availability

The author has nothing to report.
